# First description of the male *Quelaestrygon puetzi* Smetana, 1999 (Coleoptera, Staphylinidae, Staphylinini) from China

**DOI:** 10.3897/zookeys.944.53482

**Published:** 2020-06-30

**Authors:** Yanpeng Cai, Xiaoyan Li, Hongzhang Zhou

**Affiliations:** 1 Morphological Laboratory, Guizhou University of Traditional Chinese Medicine, Guiyang, 550025, Guizhou, China Guizhou University of Traditional Chinese Medicine Guiyang China; 2 Key Laboratory of Zoological Systematics and Evolution, Institute of Zoology, Chinese Academy of Sciences, 1 Beichen West Rd., Chaoyang District, Beijing 100101, China Langfang Normal University Langfang China; 3 University of the Chinese Academy of Sciences, 19A Yuquan Rd., Shijingshan District, Beijing, 100049, China Institute of Zoology, Chinese Academy of Sciences Beijing China; 4 Hebei Key Laboratory of Animal Diversity, Langfang Normal University Aiminxidao 100, Anci Area, Langfang 065000, Hebei Province, China University of the Chinese Academy of Sciences Beijing China

**Keywords:** Morphology, rove beetle, taxonomy

## Abstract

A male of the very rare and phylogenetically puzzling species, *Quelaestrygon puetzi* Smetana, 1999, is described for the first time based on a single specimen from Sichuan Province, China. High quality color images and line drawings of the male external and genitalic traits are provided.

## Introduction

The monotypic genus *Quelaestrygon* Smetana, 1999 (Staphylinidae, Staphylininae, Staphylinini) was established based on two female specimens of *Quelaestrygon puetzi* Smetana, 1999 collected from the mountain areas in Sichuan, China.

Initially, this distinctive genus was assigned to the subtribe Quediina mostly due to its overall similarity to the other members of the subtribe. Later, a series of phylogenetic studies of Staphylinini were successively conducted by various authors (e.g., Solodovnikov 2006; Solodovnikov and Schomann 2009; Chatzimanolis et al. 2010; Brunke and Solodovnikov 2013; Brunke et al. 2016; Żyła and Solodovnikov 2019; Tihelka et al. 2020). As a result, the subtribe Quediina was confirmed to be highly polyphyletic and redefined as a more restricted group, while many genera were moved out from Quediina sensu Brunke et al. (2016). The systematic position of some of them, including *Quelaestrygon* Smetana, unfortunately, is still unclear (Brunke et al. 2016).

**Figure 1.**
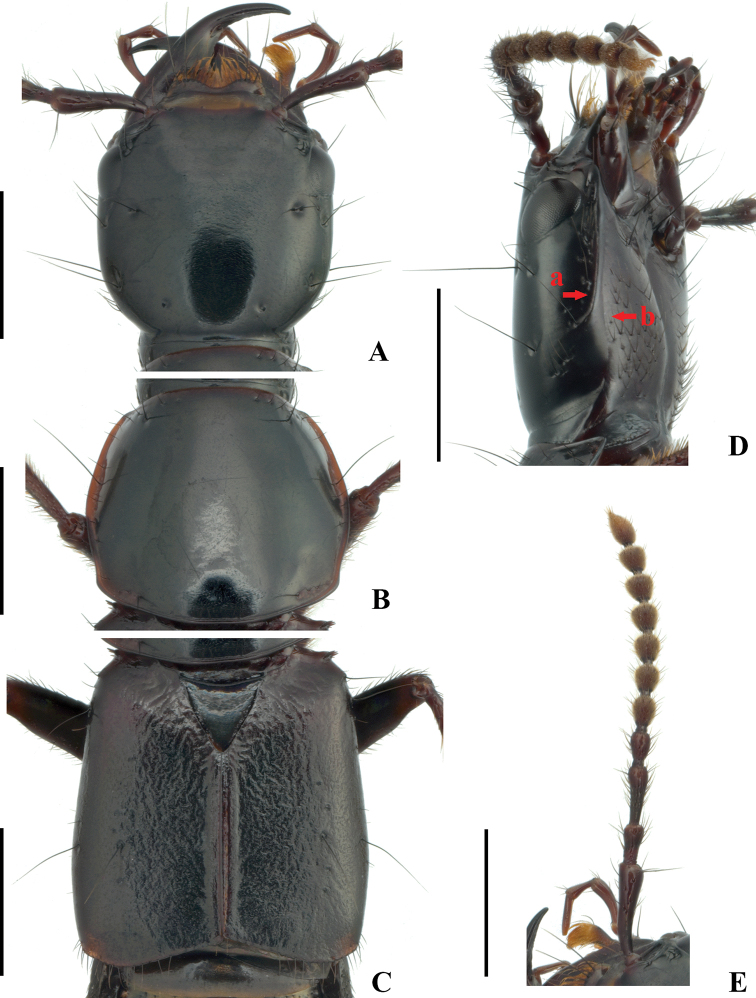
Male of *Quelaestrygon puetzi* Smetana **A** head in dorsal view **B** pronotum in dorsal view **C** elytra in dorsal view, showing sculpture **D** head in lateral view, showing postmandibular ridge (a) and infraorbital ridge (b) **E** antenna, detail. Scale bars: 2.00 mm.

**Figure 2.**
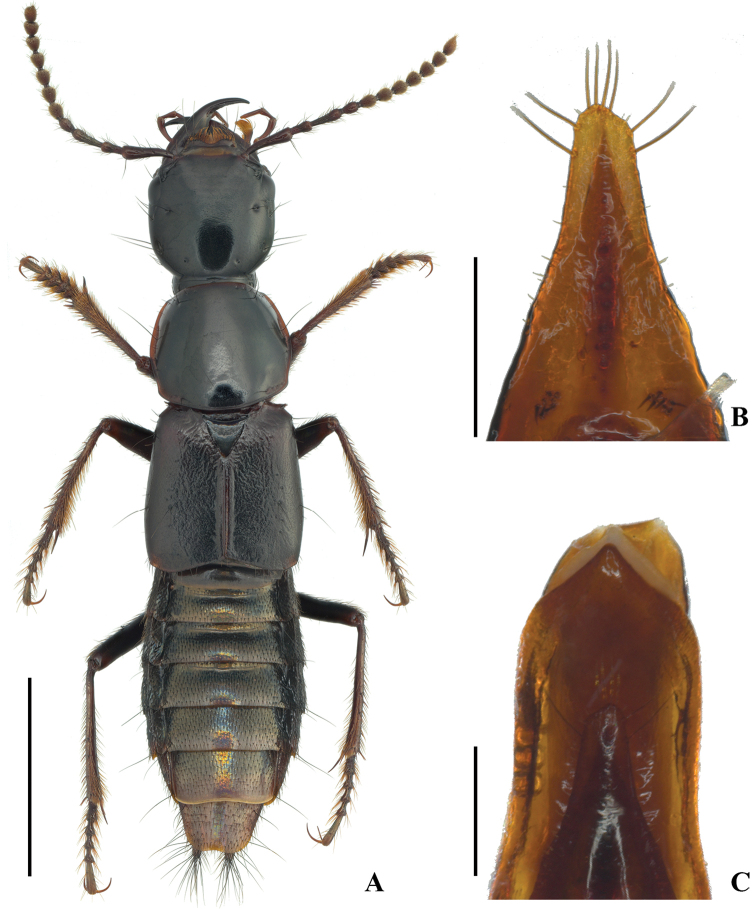
Male of *Quelaestrygon puetzi* Smetana, morphology **A** body in dorsal view **B** aedeagus, underside of paramere **C** aedeagus, apical portion of median lobe, parameral view. Scale bars: 5.00 mm (**A**), 0.50 mm (**B, C**).

This peculiar genus is mainly characterized by its large size and the long appendages (Fig. 2A), the simultaneous presence of the postmandibular and infraorbital ridges on the head (Fig. 1D), the distinct basolateral mandibular ridge, removed from the lateral mandibular margin and bordered by a deep and very wide depression (Fig. 1A), the first four antennal segments devoid of dense appressed pubescence (Fig. 1E), the absence of dorsal rows of punctures on the pronotum (Fig. 1B), and the surface sculpture of the elytra with fine leather-like rugulae and scratch-like short lines, lacking the usual punctation and pubescence (Fig. 1C) (Smetana 1999).

Examination of rove beetle specimens collected from Sichuan Province, China revealed a male of this rare species. The aim of this study was to describe the male of *Q. puetzi* Smetana, in the hope that the new information provided could help with future resolution of this taxonomically uncertain genus.

## Material and methods

The male specimen was relaxed in warm water (60 °C) for 5–8 hours for dissection of the abdominal segments VIII–X and the genitalia. After examination, the dissected body parts were glued back to the mounting card for future study. Observation, dissection and measurements were performed using a stereo microscope (Zeiss SteREO Discovery V20). Images of the adult and genitalia were captured with an AxioCam MRc 5 camera attached to a Zeiss Axio ZoomV16 Fluorescence Stereo Zoom Microscope, and photomontage was performed in Zen 2012 (blue edition) imaging software. Inkscape V0.91 was used to make the line drawings. The abdominal tergites and sternites were entirely flattened for the line drawings to make the illustrations more comparable among species.

The specimen examined was deposited in the Institute of Zoology, Chinese Academy of Sciences (**IZ-CAS**).

Morphological terminology followed Smetana (1999), Smetana and Davies (2000).

The following abbreviations are used in the text:

BL body length (from apex of clypeus to apex of abdominal tergite VIII);

BW body width (maximal body width, usually equal to EW);

HL head length (from base of clypeus to neck constriction);

HW head width (maximal head width, including eyes);

PL pronotal length (along midline of pronotum);

PW pronotal width (maximal pronotal width);

EL elytral length (maximal elytral length);

EW elytral width (maximal elytral width);

ESL elytral suture length (from apex of scutellum to apex of elytral suture);

AW abdominal width (maximal width of abdomen);

HEL (head) eye length;

HTL (head) temporal length.

## Taxonomy

### Quelaestrygon

Taxon classificationAnimaliaColeopteraStaphylinidae

Smetana, 1999: 241.

B428CCD8-537A-543D-8DB0-9B60AFEBEDD3

#### Type species.

*Quelaestrygon puetzi* Smetana, 1999, by monotypy.

### Quelaestrygon puetzi

Taxon classificationAnimaliaColeopteraStaphylinidae

Smetana, 1999

0F20ECF5-93DC-5BBA-8816-FB58E558F7B6

Figs 1 , 2 , 3

Quelaestrygon puetzi Smetana, 1999: 246 (type locality: China: Sichuan, Daxue Shan Gongga Shan, Mt. Hailuogou Glacier Park, 2620–1940 m).

#### Material examined.

1 ♂; China, Sichuan Province, Mt. Emei, Leidongping; 8. VI. 2014; Chengbin Wang leg.

#### Measurements.

BL = 18.3 mm, BW = 4.1 mm, HL/PL/EL = 1.00: 0.96: 1.28, HW/PW/EW/AW = 1.00: 1.13: 1.28: 1.32, HW/HL = 0.94, HEL/HTL = 0.39, PW/PL = 1.11, EW/EL = 0.95, ESL/EL = 0.59.

#### Description of female.

See Smetana (1999: 246–247).

#### Description of male.

**Figure 3.**
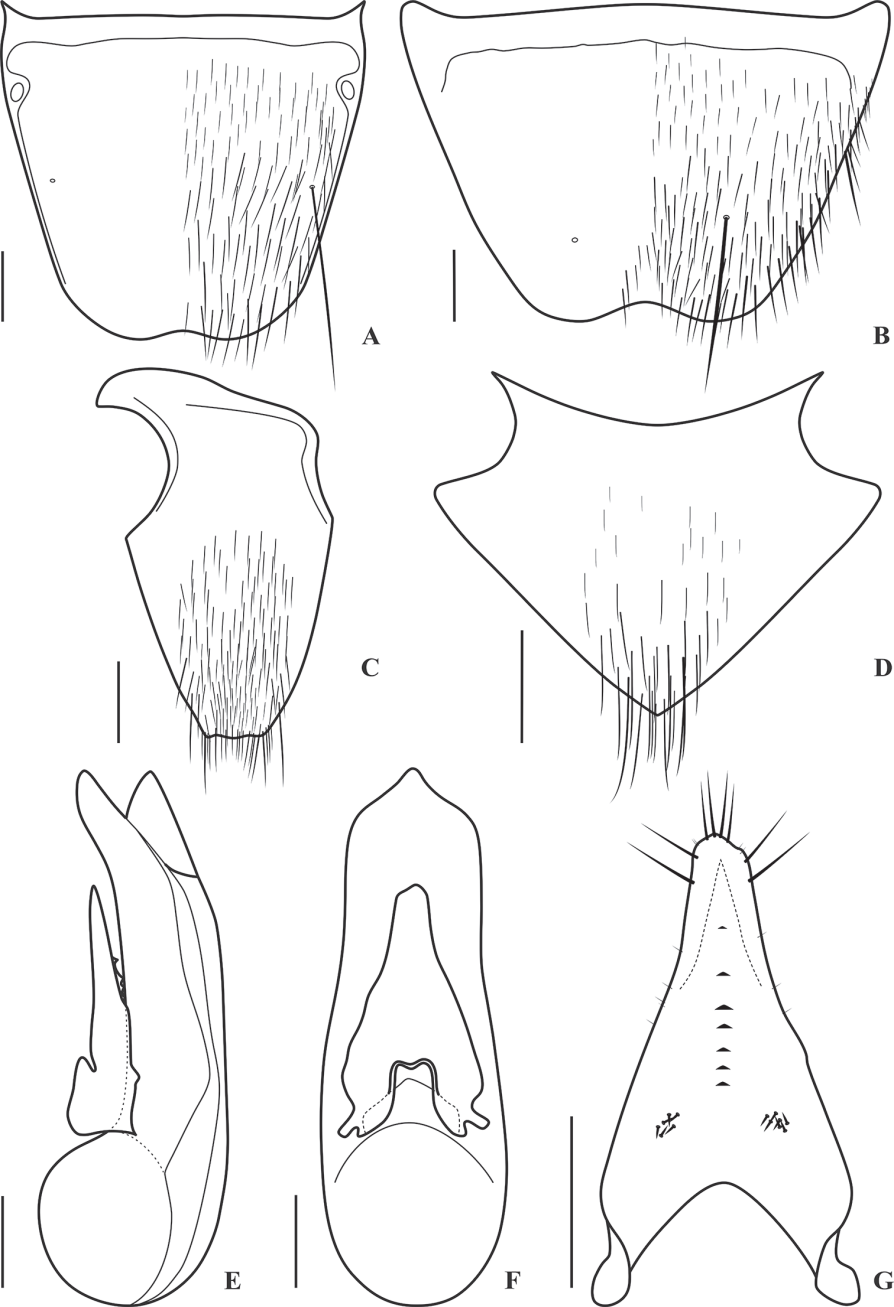
Male terminalia and genitalia of *Quelaestrygon puetzi* Smetana **A** male tergite VIII **B** male sternite VIII **C** male sternite IX **D** male tergite X **E** aedeagus, lateral view **F** aedeagus, parameral view **G** aedeagus, underside of paramere. Scale bars: 0.50 mm.

Male with first four segments of foretarsus strongly dilated, sub-bilobed, each heavily covered with tenent setae ventrally, segment II slightly wider than apex of tibia; tergite VIII (Fig. 3A) with basal ridge complete, nearly straight, with one long seta on each side, apical margin with shallow and narrow medioapical emargination; sternite VIII (Fig. 3B) with basal ridge complete, slightly sinuate, with one long seta on each side, apical margin with shallow and wide medioapical emargination, a very small triangular area in front of the emargination impunctate; sternite IX (Fig. 3C) with basal portion short and wide, apex almost truncate, apical margin forming indistinct M-shaped indention; tergite X (Fig. 3D) with basal side broadly and shallowly concave, apical margin complete, forming a right angle; aedeagus robust and strongly sclerotized, in lateral view (Fig. 3E) with apex of paramere distinctly not reaching that of median lobe, apical 1/4 of median lobe strongly bent toward parameral side, without any process at apex; aedeagus in parameral view (Fig. 3F) with paramere distinctly narrower than median lobe, wide at base, then gradually narrowed into obtuse apex, forming a near triangle shape, median lobe parallel-sided laterally, distinctly constricted at apex, apex somewhat pointed (Figs 2C, 3F); apical portion of paramere with four moderately long apical setae, and two similar subapical setae on each lateral side below apex, underside with seven small tooth-shaped processes along the more strongly sclerotized and pigmented midline, and 6–7 spike-like sensory peg setae arranged in cluster far below apex on each side (Figs 2B, 3G).

#### Distribution.

*Quelaestrygon puetzi* Smetana is at present known only from several mountain areas in Sichuan Province of China: Mt. Gongga, Mt. Jinping and Mt. Emei. The examined male specimen was hand-collected at a parking lot after landing on the collector’s clothes.

## Supplementary Material

XML Treatment for Quelaestrygon

XML Treatment for Quelaestrygon puetzi
